# Identification and validation of miR‐29b‐3p and LIN7A as important diagnostic markers for bone non‐union by WGCNA

**DOI:** 10.1111/jcmm.18522

**Published:** 2024-07-03

**Authors:** Guojian Jian, Desheng Xie, Ximu Kuang, Peihuang Zheng, Haoyuan Liu, Xuehong Dong

**Affiliations:** ^1^ Department of Orthopedics Chenggong Hospital of Xiamen University (the 73th Group Military Hospital of People's Liberation Army) Xiamen Fujian China

**Keywords:** biomarker, bone non‐union, LIN7A, miRNA‐29b‐3p

## Abstract

Bone non‐union is a common fracture complication that can severely impact patient outcomes, yet its mechanism is not fully understood. This study used differential analysis and weighted co‐expression network analysis (WGCNA) to identify susceptibility modules and hub genes associated with fracture healing. Two datasets, GSE125289 and GSE213891, were downloaded from the GEO website, and differentially expressed miRNAs and genes were analysed and used to construct the WGCNA network. Gene ontology (GO) analysis of the differentially expressed genes showed enrichment in cytokine and inflammatory factor secretion, phagocytosis, and trans‐Golgi network regulation pathways. Using bioinformatic site prediction and crossover gene search, miR‐29b‐3p was identified as a regulator of LIN7A expression that may negatively affect fracture healing. Potential miRNA‐mRNA interactions in the bone non‐union mechanism were explored, and miRNA‐29‐3p and LIN7A were identified as biomarkers of skeletal non‐union. The expression of miRNA‐29b‐3p and LIN7A was verified in blood samples from patients with fracture non‐union using qRT‐PCR and ELISA. Overall, this study identified characteristic modules and key genes associated with fracture non‐union and provided insight into its molecular mechanisms. Downregulated miRNA‐29b‐3p was found to downregulate LIN7A protein expression, which may affect the healing process after fracture in patients with bone non‐union. These findings may serve as a prognostic biomarker and potential therapeutic target for bone non‐union.

## 1 INTRODUCTION

Fracture healing is a complex biological process (BP) involving inflammation, regenerative healing and bone remodelling.^1^, ^2^ Most fractures normally heal within 6–8 weeks. However, incomplete healing environments can result in delayed or non‐union fractures, which are defined as fractures that have not healed within 9 months and have shown no signs of healing for three consecutive months.^3^, ^4^ The prevalence of non‐union in patients with fractures is 5%–10%.^5^, ^6^ The primary conventional risk factors associated with non‐union or delayed healing of fractures include advanced age, female gender,^7^ smoking^8^ and diabetes.^9^ However, reliance on these factors alone for prediction has proven to be insufficiently accurate, making it difficult to effectively guide early interventions to prevent non‐union.

MicroRNAs (miRNAs) are short, non‐coding, single‐stranded RNAs that regulate gene expression and play a critical role in precision medicine in various fields. Many miRNAs have been reported to act as regulators of the pathogenesis of bone non‐union.^10^ For example, miRNA‐133a inhibits fracture healing by targeting RUNX2/BMP2.^11^ Some researchers have also found that the N6‐methyladenosine modification of miR‐7212‐5p, facilitated by methyltransferase‐like 3, stimulates osteoblast differentiation and contributes to the process of fracture healing.^12^ Furthermore, the investigation revealed that exosomal miR‐25 derived from bone marrow mesenchymal stem cells promotes fracture healing in mice by modulating the ubiquitination and degradation of Runx2 via SMURF1.^13^ The biological molecular mechanisms by which miRNAs affect gene expression and regulate bone non‐union pathogenesis are not fully understood and require further in‐depth studies for improved diagnosis and treatment. The role of the miRNA‐29 family in cellular and stromal changes in osteoarthritis is well established,^14^ but its impact on bone non‐union is unclear.

Weighted gene co‐expression network analysis (WGCNA) is a powerful tool for analysing the relationship between modular genes, rather than individual genes, and robust gene selection methods.^15^, ^16^ This method is useful for studying genes associated with clinical traits and identifying biomarkers in diseases such as oncology,^17^, ^18^ immune disorders,^19^ and chronic obstructive pulmonary disease (COPD).^20^ In this study, we analysed trait‐related miRNAs and genes in patients with bone non‐union using WGCNA, performed biological‐functional correlation analysis, identified the biomarker gene LIN7A, also known as Lin‐7 homologue A, and analysed the specific sites of miR‐29b‐3p regulating LIN7A by database prediction. LIN7A is a member of the Lin‐7 family, which plays important roles in the regulation of cell polarity, protein trafficking, ^21^, ^22^, ^23^, ^24^LIN7A is widely expressed in various tissues, including the brain, heart, liver and kidney.^25^, ^26^ However, the expression and function of LIN7A in bone non‐union is not well understood. Finally, we verified the expression levels of miR‐29b‐3p and LIN7A in blood samples from patients with bone non‐union at a local hospital. We found that LIN7A can be affected by miRNA‐29b‐3p, resulting in skeletal non‐healing. Together, this research improved our understanding of the causes and basic molecular mechanisms of bone non‐union and thus provide valuable insights into its pathogenesis.

## 2 MATERIALS AND METHODS

### 2.1 Differential expression analysis in the GEO matrix

In this study, we used the “limma” package in R for data quality control, processing and statistical analysis.^27^ The gene expression profiles were normalized using the multi‐array average (RMA) method. Patients were categorized into fracture healing and non‐union groups based on clinical characteristics. To identify differentially expressed miRNAs and genes (DEGs), we used a significance threshold of adjusted *p*‐value <0.05 and |log_2_ fold change| ≥1.

### 2.2 Identification of non‐union‐related genes by weighted gene co‐expression network analysis

We used the WGCNA package to create a gene co‐expression network suitable for identifying genes associated with specific phenotypic modules that are not associated with non‐union.^15^, ^16^, ^28^, ^29^ Our methodology involved calculating the Pearson correlation coefficient (PCC) for all gene pairs to create an adjacency matrix, which was then transformed into a topological overlap matrix (TOM). Using average linkage hierarchical clustering, we grouped genes with similar characteristics into modules. We determined gene significance (GS) and module membership (MM) to associate modules with clinical traits. Finally, we visualized the co‐expression network of modules and used module‐associated genes for subsequent analysis.

### 2.3 Gene ontology functional annotation

To further understand the biological functions of DEGs and module‐related DEGs in non‐union, we performed GO functional enrichment analysis using the “clusterProfiler” R package.^30^ The thresholds were *p* < 0.05 and FDR <0.05.

### 2.4 Screening of hub‐related genes

To identify miRNAs and genes associated with fracture non‐union, we performed co‐expression network analysis and used Venn plots to intersect highly module‐related miRNAs with differentially enriched miRNAs. This allowed us to obtain differentially expressed trait‐related module miRNAs associated with fracture non‐union. We then extracted the expression profiles of hub miRNA genes from the non‐union GEO matrices and used miRDB and TargetScan online databases to predict genes that may interact with differential miRNAs. To map the correlation of these genes, we used Cytoscape software.^31^ In Cytoscape, the expression correlation application, used for gene correlation mapping and network analysis, allows for the computation and visualization of gene expression correlations between different conditions or samples. During the process of creating the visual representation, the parameters employed included a label font size of 12, label colour set to black, and a node size of 35. Finally, we intersected predictive genes with highly module‐related DEGs to obtain differentially expressed trait‐related module DEGs associated with fracture non‐union.

### 2.5 Luciferase reporter assay

Luciferase reporter assays were performed as previously described.^32^ HER293T cells were transfected with pCMV5‐mi‐29b‐3p, pGL3‐LIN7A‐Luc reporter constructs (Promega), and a Renilla luciferase vector (Promega). Luciferase activity was measured according to the manufacturer's instructions (Promega). Renilla activity was used as a control for transfection efficiency. Each experiment was performed in triplicate to ensure biological reproducibility.

### 2.6 Validation of hub genes expression and quantitative real‐time PCR analysis

To evaluate the expression levels of biomarker genes in patients, blood samples were collected from two groups: fracture healing (*n* = 5) and non‐union (*n* = 5) at the Department of Orthopaedics, Chenggong Hospital of Xiamen University (the 73th Group Military Hospital of People's Liberation Army), Xiamen, Fujian, China. Red blood cells were lysed using red blood cell lysis solution (Solarbio, China), and cell precipitates were collected by centrifugation at 450 rpm for 10 min. The precipitates were further lysed with TRIzol reagent (TIANGEN, China), and RNA was extracted and purified using chloroform, isopropanol and ethanol solutions. Complementary DNA (cDNA) was synthesized using either the TaqMan MicroRNA Reverse Transcription Kit (Applied Biosystems, Foster City, CA) or the PrimeScript Reverse Transcriptase Reagent Kit (Takara, Osaka, Japan). To normalize the expression levels of miR‐29b‐3p and LIN7A, U6 or glyceraldehyde‐3‐phosphate dehydrogenase (GAPDH) was used as an internal control, and a 2^−ΔΔCt^ value was obtained for relative expression. Taqman probes (2435 for miR‐29b‐3p, 1973 for U6 (Applied Biosystems)) were used for qRT‐PCR detection. The other primers used were as follows: LIN7A sequence of forward primer and reverse primer: 5′‐GCAACAGCAAAGGCAACAGT‐3′ and 5′‐CTCTTTTGAGGCCTCCGTGT‐3′ and GAPDH sequence of forward primer and reverse primer: 5′‐CTGGGCTACACTGAGCACC‐3′ and 5′‐AAGTGGTCGTTGAGGGCAATG‐3′.

### 2.7 Statistical analysis

The data were analysed using R 4.1.2 software and Cytoscape 3.7.1 software, and the data were analysed and plotted using GraphPad Prism 8. The R packages used were ‘clusterProfiler’, ‘dplyr’, ‘enrichplot’, ‘flashClust’, ‘ggplot2’, ‘ggrepel’, ‘Goplot’, ‘limma’, ‘org.Hs.eg.db’, ‘org. ggrepel’, ‘Goplot’, ‘limma’, ‘org.Hs.eg.db’, ‘stringr’, ‘tidyverse’, ‘WGCNA’. *p* values were adjusted using the Benjamini & Hochberg (BH) method, and a *p* value less than 0.05 was considered statistically significant unless otherwise noted. Quantitative data (mean ± SEM) were subjected to the Student *t* test, using GraphPad Prism 8 software (GraphPad Software, San Diego, CA).

## 3 RESULTS

### 3.1 Data download and differential expression analysis

**FIGURE 1:**
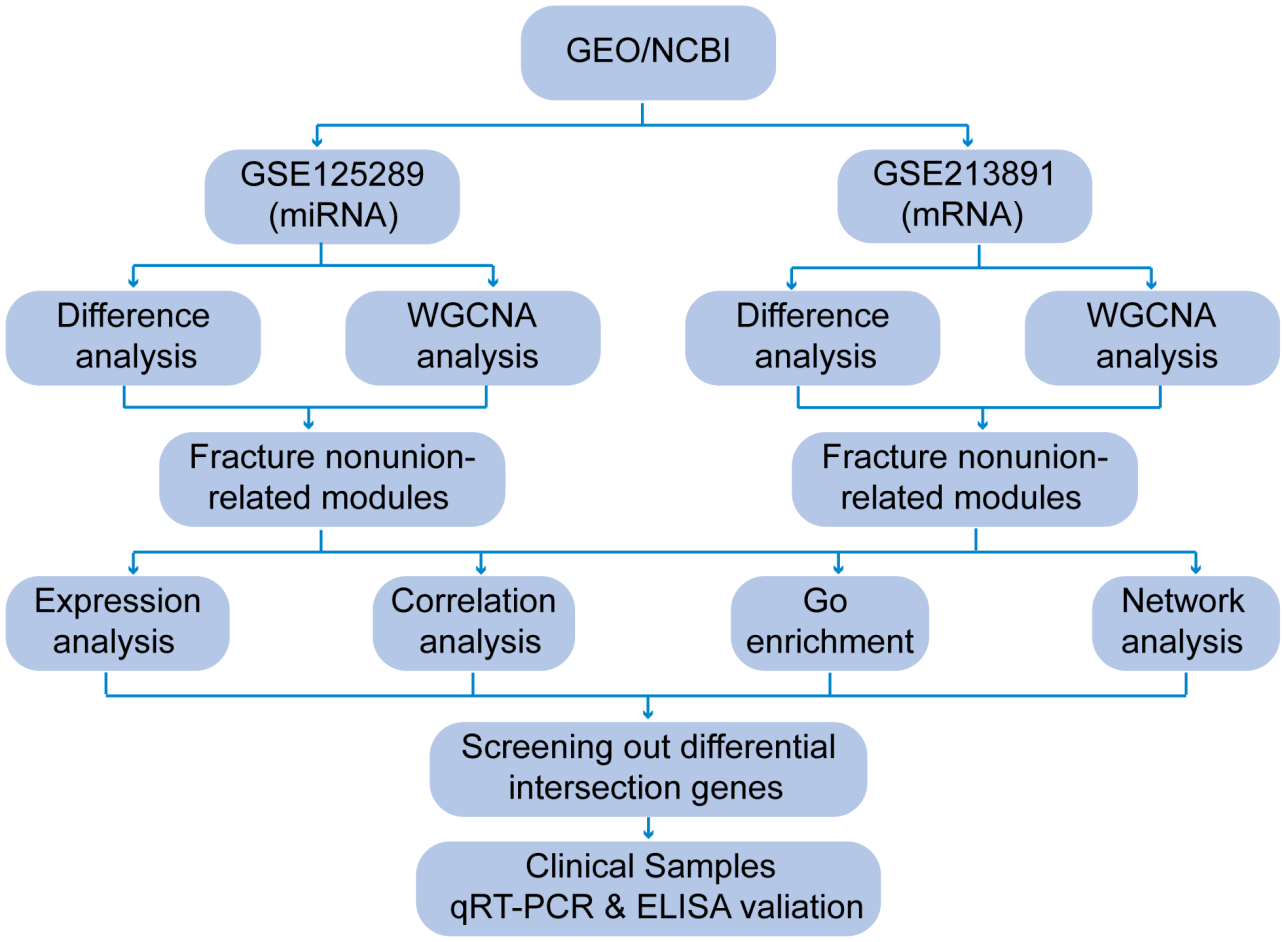
Flow chart of the whole process in this study.

To explore the potential relationship between miRNAs and mRNAs in fracture non‐union, we systematically searched the gene expression omnibus (GEO) database (https://www.ncbi.nlm.nih.gov/gds/) for relevant high‐throughput functional genomic expression matrices, a database containing post‐fracture non‐union comprehensive database of data. During the mRNA expression data selection process, several inclusion criteria were developed: (1) the species studied was Homo sapiens; (2) the sample types in the relevant data matrices that would be analysed identically were consistent; and (3) all data were publicly available and usable. Finally, one miRNA matrix and one mRNA matrix were selected for the next step of the analysis. The overall procedure of this study is shown in Figure 1.

We downloaded two datasets, GSE125289 and GSE213891, from the GEO database containing miRNA and gene expression data relevant to our study. The GSE125289 matrix consists of miRNA expression data from bone tissue at the fracture ends of patients with non‐union infected fractures and newly closed fractures. The GSE213891 matrix contains miRNA and gene expression data from tissues of patients with non‐union and healing fractures.

**FIGURE 2:**
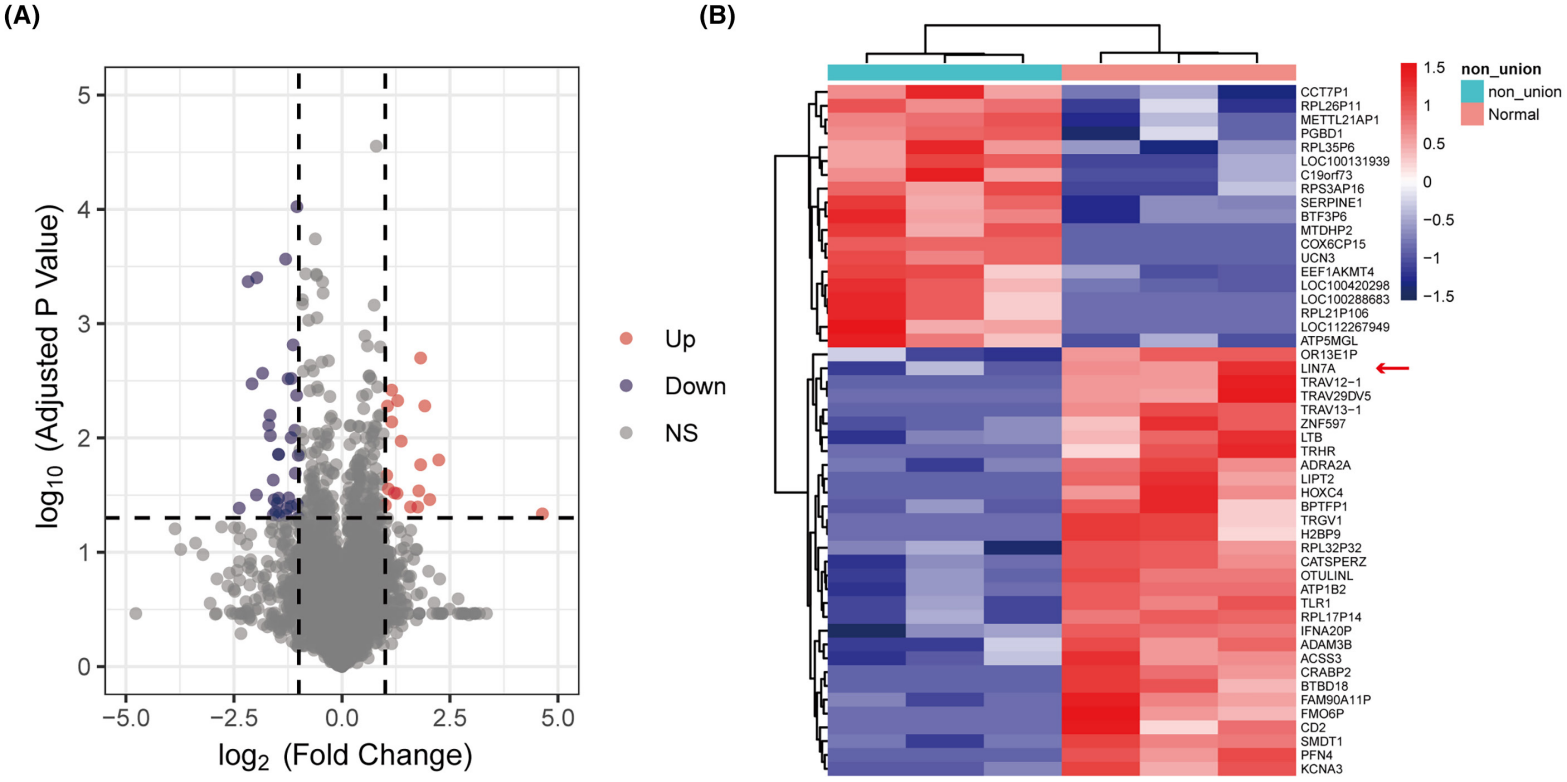
Identification of differentially expressed genes in GSE213891. (A) The volcano map of DEGs in the non‐union expression matrix. (B) Heatmap of the over‐expressed and low‐expressed genes.

We analysed the data using the limma package and selected miRNAs and genes with a |log_2_ fold change| ≥1 and adjusted *p*‐value <0.05 as screened differential change miRNAs and DEGs. We identified five fracture non‐union associated miRNAs in the GSE125289 matrix and plotted them in volcano plots to compare their expression between tissues of patients with non‐union and healing fractures (Figure S1). A complete list of differential miRNAs in patients with non‐union fractures can be found in Table S1. In addition, analysis of the GSE213891 matrix identified 54 genes associated with non‐union fractures, of which 20 were upregulated and 34 were downregulated. We generated volcano plots to compare the expression of DEGs between fracture non‐union and fracture healing patient tissues (Figure 2A) and used heat maps to show the genes with the largest differences in up and downregulated expression (Figure 2B). A complete list of all DEGs can be found in Table S2.

### 3.2 Identification of miRNAs in bone non‐union and construction of WGCNA network

**FIGURE 3:**
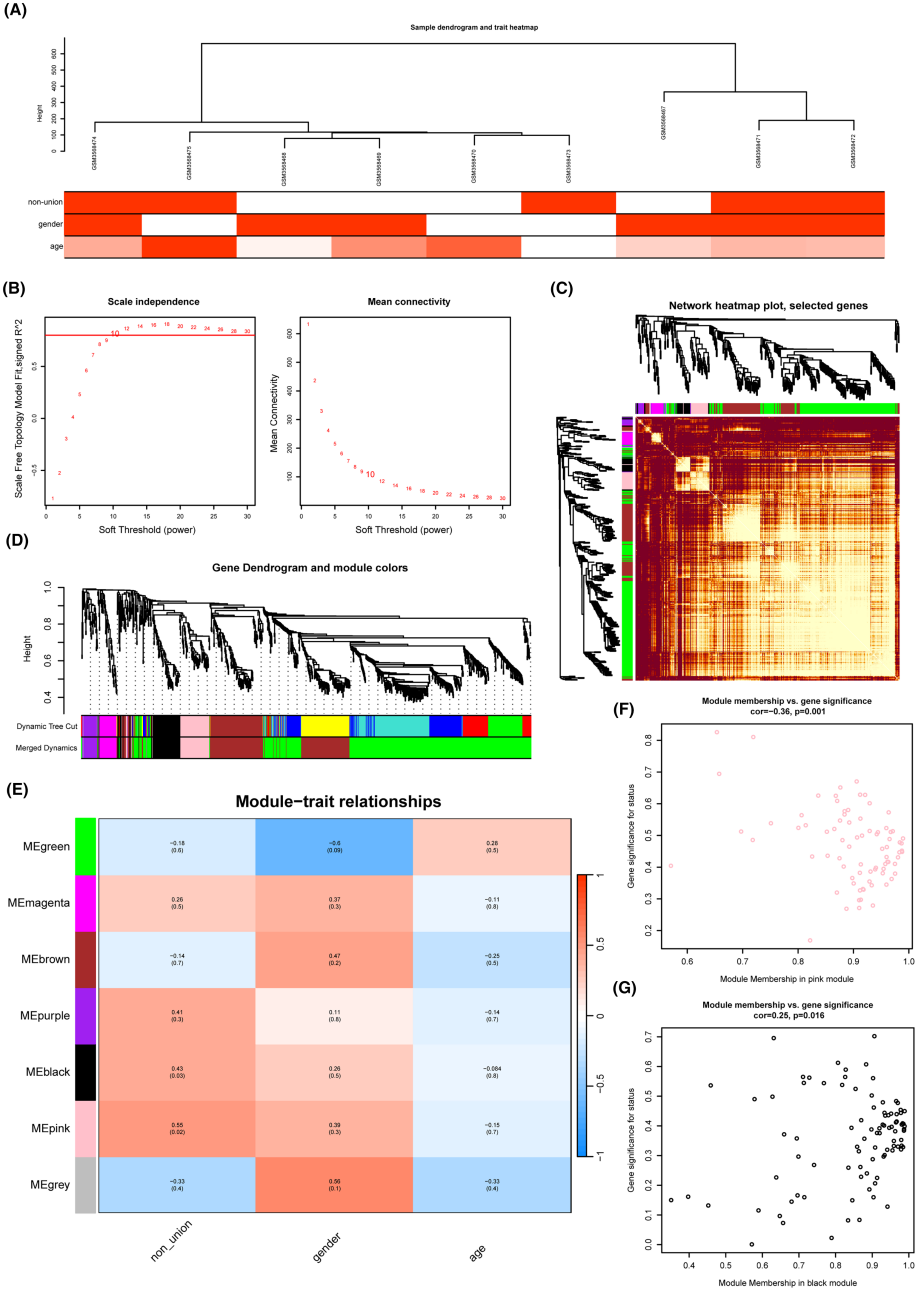
The construction of WGCNA network in the fracture non‐union matrix GSE125289. (A) Filtering of outliers in the fracture non‐union matrix. (B) The selection of soft threshold during the WGCNA construction. (C) Topological overlap matrix (TOM) heatmap of all genes in the analysis. (D) Dendrogram of all expressed genes clustered based on a dissimilarity measure in non‐union. (E) Heatmap of the correlation between module eigengenes and clinical traits in non‐union. (F) Scatter plots of the degree and *p*‐value of Cox regression in MEpink module. (G) Scatter plots of the degree and *p*‐value of Cox regression in MEblack module.

The nine samples from the GSE125289 dataset were analysed using the hclust function, and the results showed that the samples were well clustered with no outlier samples (Figure S2A). Furthermore, the samples were clustered in combination with clinical data and phenotypic heat map analysis (Figure 3A). The miRNAs with the top 5000 MAD values were selected for WGCNA, and the soft threshold was determined based on its impact on the independence and average connectivity of the WGCNA. To ensure the construction of a scale‐free network, an empirical analysis was performed to select the appropriate soft threshold value (Figure 3B). A soft threshold of 10 was chosen because it provided a stable topological model fit index and connectivity, and the connectivity among miRNAs followed the distribution of the scale‐free network (Figure 3C). The hierarchical clustering tree among miRNAs was constructed based on the TOM matrix, and the total number of merged miRNA modules was identified as 7 using dynamic tree cutting, with each module having a unique colour as an identifier (Figure 3D and Figure S2B). The purple, black and pink modules were found to have a strong correlation (Figure S2C). The relationship between each co‐expression module and clinical features was calculated, and the correlation heat map between different modules and clinical features was drawn (Figure 3E). It was found that the module with the highest correlation with fracture non‐healing was the MEPink module (coefficient of 0.55, *p*‐value 0.02), followed by the MEblack module (coefficient of 0.43, *p*‐value 0.03) (Figure 3E).

In addition, GS and MM were calculated to associate modules with clinical features (Figure S2D). The pink and black modules were identified as key modules to map the correlation between GS and MM, focusing on disease status as the main clinical feature, and these miRNAs were used to identify key miRNAs. The correlation coefficient between the pink module GS and MM was found to be cor = −0.36, *p* = 0.001 (Figure 3F), while the correlation coefficient between the black module GS and MM was cor = 0.25, *p* = 0.016 (Figure 3G). This indicates that fracture non‐healing was significantly correlated with the pink and black Modules.

### 3.3 Identification of DEGs in bone non‐union and construction of WGCNA network

**FIGURE 4:**
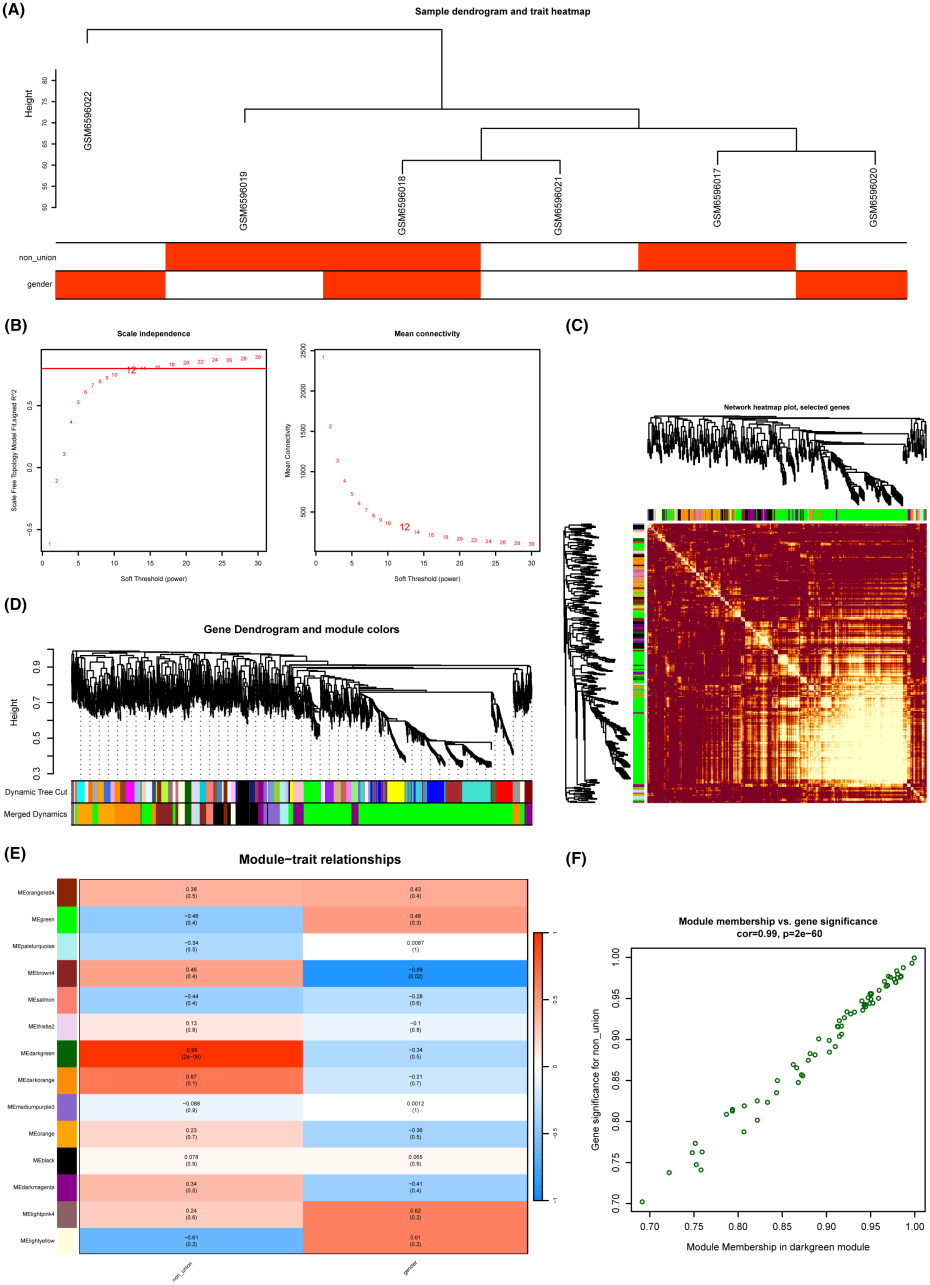
The construction of WGCNA network in the fracture non‐union matrix GSE213891. (A) Filtering of outliers in the fracture non‐union matrix. (B) The selection of soft threshold during the WGCNA construction. (C) Topological overlap matrix heatmap of all genes in the analysis. (D) Dendrogram of all expressed genes clustered based on a dissimilarity measure in non‐union. (E) Heatmap of the correlation between module eigengenes and clinical traits in non‐union. (F) Scatter plots of the degree and *p*‐value of Cox regression in MEdarkgreen module.

By analysing the GSE213891 matrix, we identified 54 genes associated with bone non‐union (Figure 2A). We also used the same approach to construct the WGCNA network. The samples were clustered together with clinical data, and phenotypic heat map analysis was performed (Figure 4A and Figure S3A). The top 5000 DEGs with the highest MAD values were used for the WGCNA analysis, and the topological model fit index and connectivity were good when the soft threshold was set to 12 (Figure 4B). The hierarchical clustering tree was constructed based on the TOM matrix, and dynamic tree cutting was used to identify a total of 14 merged DEG modules, each with a unique colour identifier (Figure 4C,D and Figure S2B), among which the darkgreen, darkorange, and mediumpurple3 modules showed a strong correlation with each other (Figure S3C).

The relationship between each co‐expression module and clinical traits was calculated, and a correlation heat map was drawn between different modules and clinical traits. Finally, trait‐related modules were identified (Figure 4E). The genes in the dark green module were found to be strongly correlated with bone non‐union, with a correlation coefficient of 0.99 and a *p* value of 2e‐06 (Figure 4E), indicating that bone non‐union is significantly associated with the dark green module.

To associate modules with clinical features, we calculated GS and MM (Figure S3D). The darkgreen module was identified as the key module, and disease status was used as the main focus of clinical features to plot GS and MM correlation to identify the key genes. The darkgreen module showed a high correlation coefficient (cor = 0.99, *p* = 2e‐60) with GS, indicating that it contains genes significantly associated with bone non‐union (Figure 4F).

### 3.4 GO functional annotation of module genes

**FIGURE 5:**
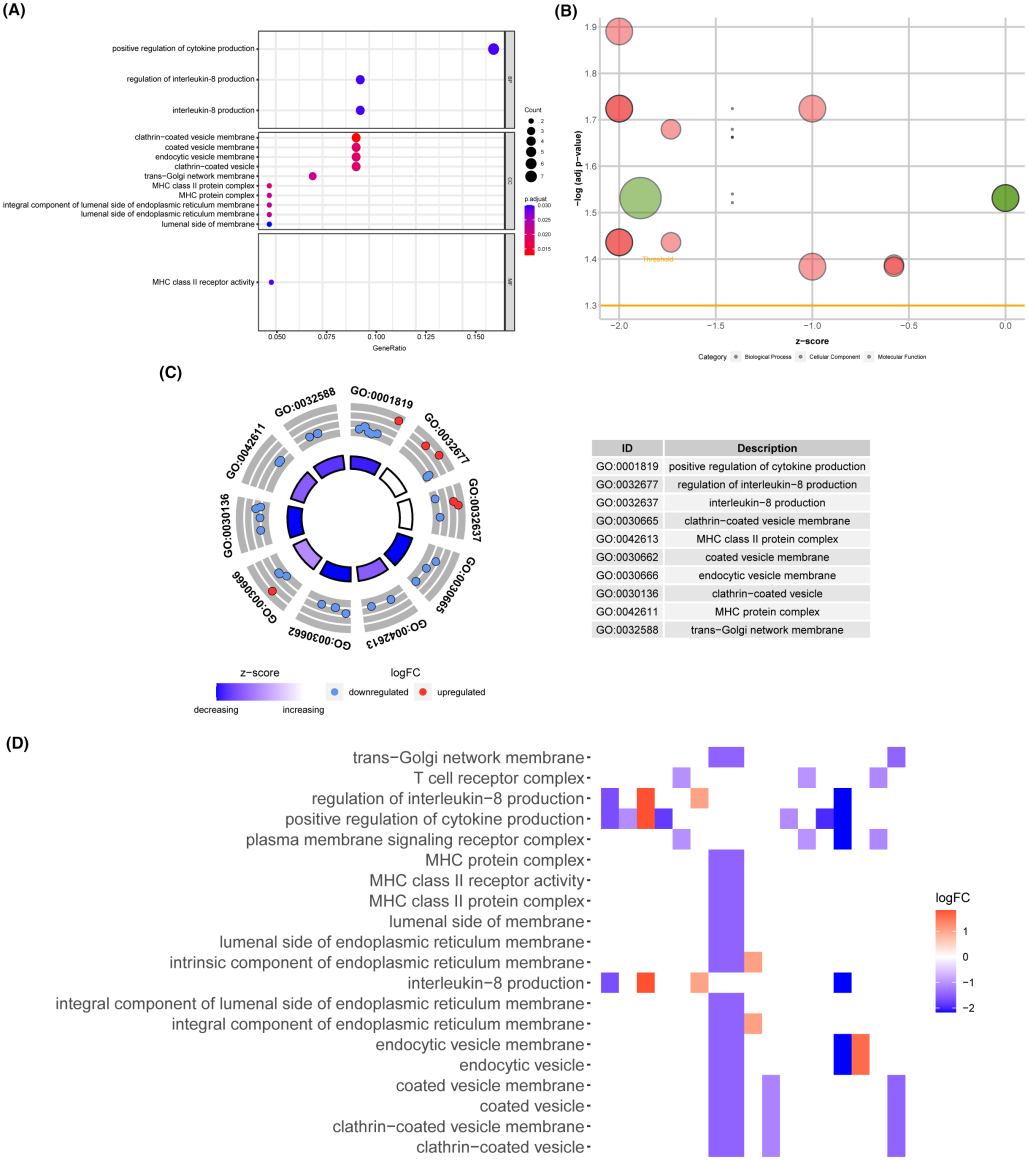
Gene ontology functional annotation of trait‐related genes and gene set enrichment analysis in GSE213891. (A) GO enrichment analysis results of characteristic genes. (B) GOBubble plot of *z*‐score calculation of enrichment pathways based on expression level of genes. (C) The outer circle presented the scatter plot of each logFC of the pathway genes. Red meant overexpression, and blue displayed decrease. (D) Heatmap of correlation between trait‐related genes and pathways.

We performed bioenrichment analysis on the genes associated with fracture non‐healing. First, we performed gene ontology (GO) analysis on the differentially expressed genes (DEGs) associated with fracture non‐healing, which revealed their association with the production of cytokines and inflammatory factors (BP), clathrin‐coated vesicle membrane (cellular component, CC), and MHC class II receptor activity (molecular function, MF) (Figure 5A). This suggests that fracture non‐healing may be related to immune cell‐mediated immunity. The CC of these genes is mainly enriched in membrane vesicle trafficking in the endoplasmic reticulum and Golgi apparatus, while the MF is mainly associated with MHC class II receptor activity. The MHC molecule is a class of proteins encoded by MHC genes that are present on the surface of all nucleated cells and whose main function is to present self or foreign antigenic peptides to T cells, thereby regulating the immune response.^33^, ^34^ Depending on their structure and function, MHC molecules can be divided into three categories: MHC class I molecules, MHC class II molecules, and MHC class III molecules.^35^, ^36^ As shown in Figure 5B, higher *z*‐scores indicate the presence of more downregulated genes enriched in the pathway. We also identified other immune‐related pathways, such as secretion of inflammatory factors, phagocytosis of endocytic vesicles, and regulation of the trans‐Golgi network (Figure 5C). In addition, the heat map shows the expression between related genes and pathways (Figure 5D). The complete GO enrichment results are shown in Table S3.

Similarly, we performed GO analysis of crossover genes between DEGs and dark green modules in fracture non‐healing, and the results revealed that these trait‐related genes for fracture non‐healing were mainly enriched in the BP and closely associated with the production of cytokines and inflammatory factors (Figure S4).

### 3.5 LIN7A was found as one of the targets for miR‐29b‐3p

**FIGURE 6:**
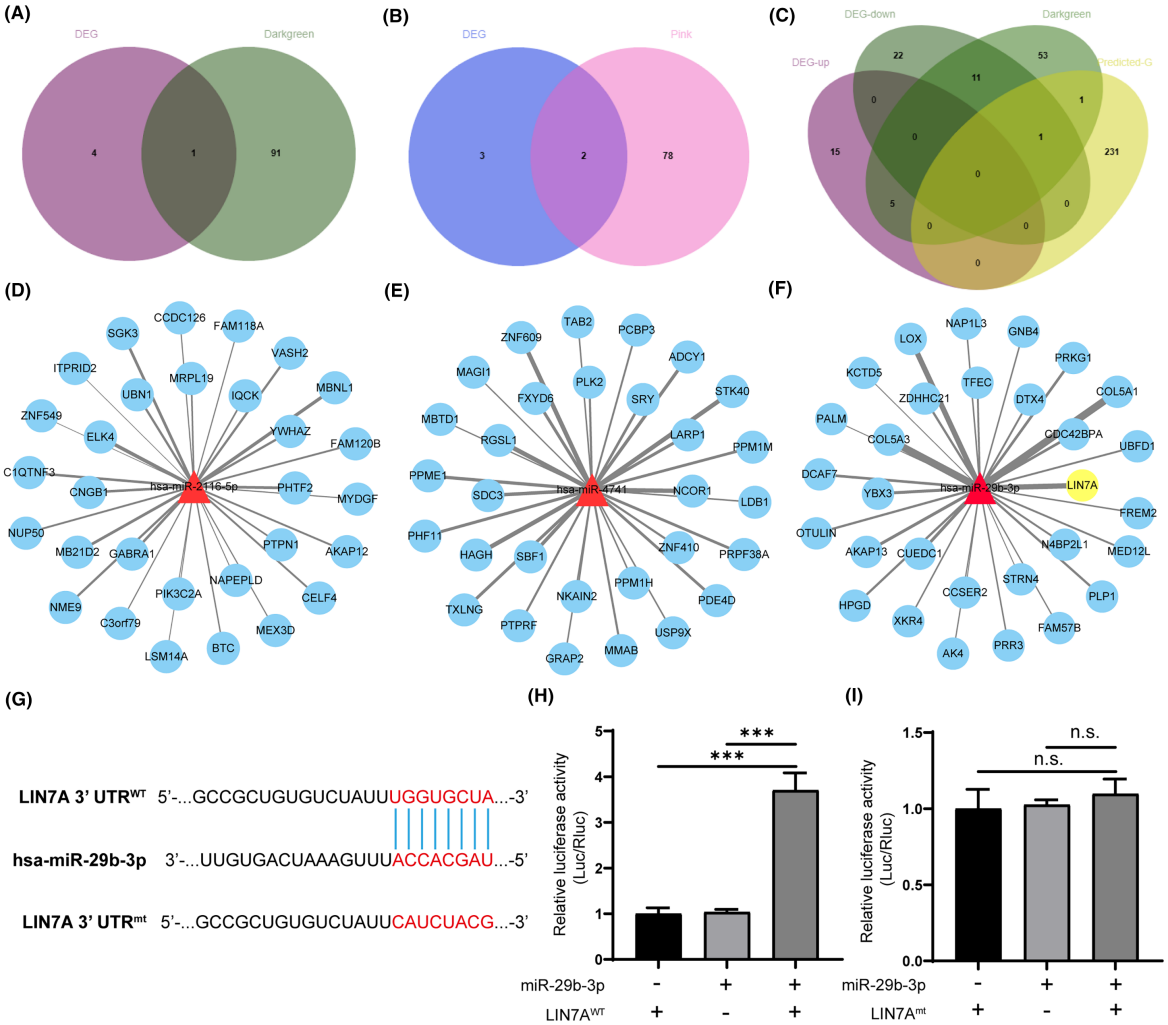
Interactions among module hub genes, genome‐wide‐associated genes and miRNAs predicted genes. (A, B) Analysis of the intersection of the differential miRNAs and pink module miRNAs (A) and black trait‐related module (B), respectively. (C) Analysis of the crossover genes with DEGs, dark green module genes, and mi‐29b‐3p predicted genes. (D–F) The interaction genes of miR‐29b‐3p (D), miR‐2116‐5p (E), and miR‐4741 (F), and constructed mRNA‐miRNA regulatory networks based on 30 prediction genes. (G) The complementary sequence of miR‐29b‐3p was present in the 3′‐UTR of LIN7A mRNA. (H) The luciferase activity of the wild‐type (wt) structure of the LIN7A 3′‐UTR. (I) The luciferase activity of the mutant (mt) structure of the LIN7A 3′‐UTR. Data represent the mean ± SEM (three independently repeated experiments); ***p < 0.001; n.s., not significant (Student *t* test).

To identify the potential molecular mechanism of miRNA regulation in bone non‐union, we used differential miRNAs and analysed cross miRNAs with pink and black trait‐related modules, respectively. We found two key miRNAs, has‐miR‐29b‐3p and has‐miR‐2116‐5p, in the pink module, while one key miRNA, has‐miR‐4741, was identified in the black module (Figure 6A,B). The three key miRNAs were then entered into the miRDB database to predict target genes with a score above 80. The analysis showed that miR‐29b‐3p predicted 447 possible regulated genes (Table S4), has‐miR‐2116‐5p predicted 149 possible regulated genes (Table S5), and has‐miR‐4741 predicted 53 potentially regulated genes (Table S6). We used Cytoscape software to predict the interaction genes of miR‐29b‐3p (Figure 6D), miR‐2116‐5p (Figure 6E), and miR‐4741 (Figure 6F), and constructed mRNA‐miRNA regulatory networks based on 30 prediction genes.

To further identify miRNA regulatory genes, we analysed the crossover genes with DEGs, dark green module genes, and miRNA predicted genes, and plotted the Venn diagram (Figure 6C, Figures S5A,B). The results showed that only LIN7A gene was present in all three gene sets, and it was predicted by miR‐29b‐3p (Figure 6C). These results suggest that miR‐29b‐3p may regulate both the expression and function of LIN7A gene and play a critical role in the pathogenesis of fracture non‐union. However, the role of LIN7A in patients with bone non‐union remains unknown.

To investigate the potential mechanism of how miR‐29b‐3p exerts its functional role, we identified LIN7A as the target gene of miR‐29b‐3p using the target prediction algorithm TargetScan (http://www.targetscan.org). The analysis revealed that the complementary sequence of miR‐29b‐3p was present in the 3′‐UTR of LIN7A mRNA (Figure 6G). To confirm that LIN7A is a direct target of miR‐29b‐3p, we performed a luciferase reporter gene assay. The results showed that overexpression of miR‐29b‐3p increased the luciferase activity of the wild‐type (wt) structure of the LIN7A 3′‐UTR (Figure 6H). However, the luciferase activity of the mutant (mt) LIN7A 3′‐UTR did not change significantly after miR‐29b‐3p expression regulation (Figure 6I). Thus, our results suggest that LIN7A may be a direct downstream target of miR‐29b‐3p in patients with bone non‐union.

### 3.6 Validation of gene expression levels by qRT‐PCR

**FIGURE 7:**
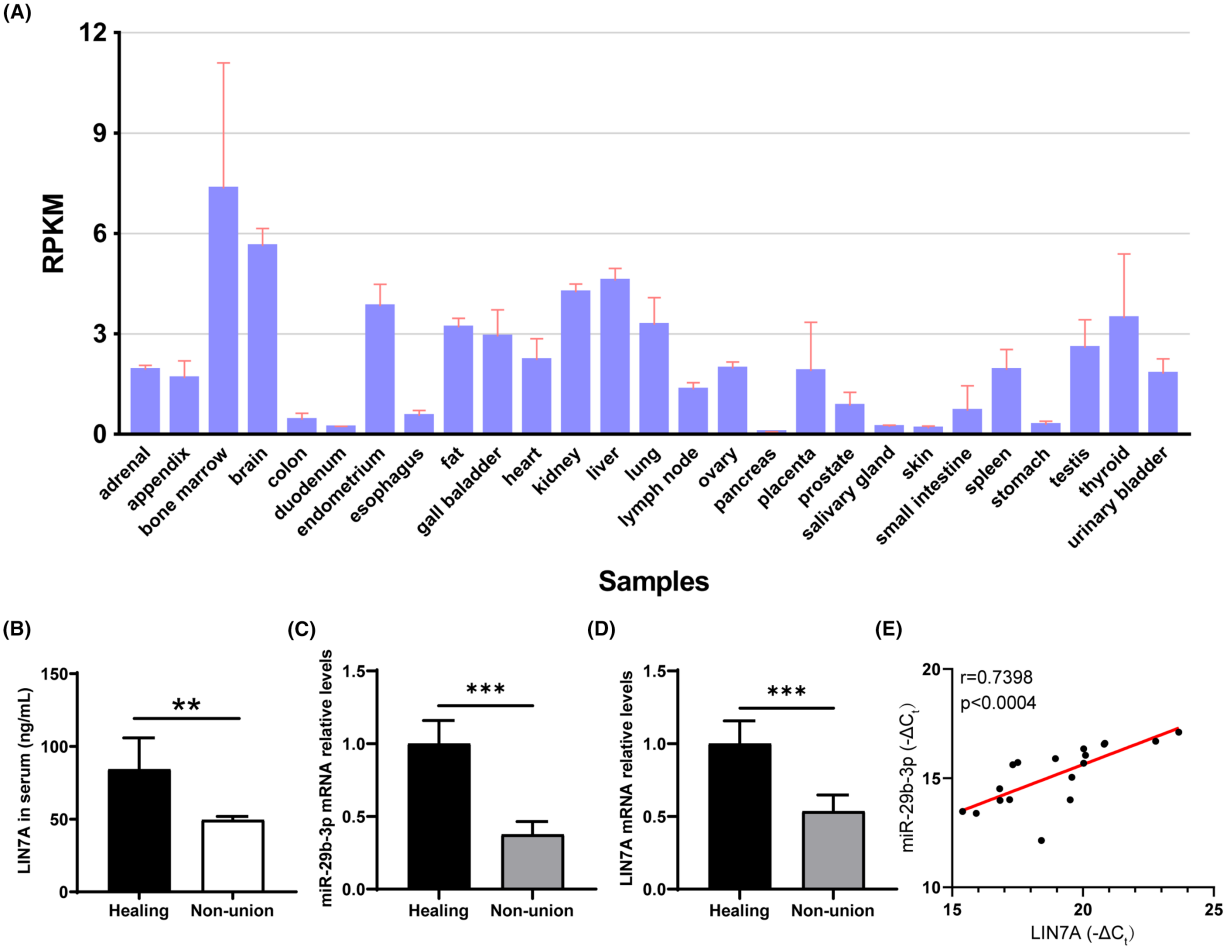
Validation of gene expression levels. (A) The expression of LIN7A in different tissues based on NCBI database. (B) RT‐qPCR analysis of LIN7A in patients with bone non‐union. (C) The transcript levels of miR‐29b‐3p in patients with bone non‐union. (D) To analyse the protein levels of LIN7A in patients' serum by ELISA. (E) The correlation analysis between LIN7A mRNA levels and miR‐501‐3p expression in non‐union patients. Data represent the mean ± SEM (three independently repeated experiments); **p < 0.01; ***p < 0.001 (Student *t* test).

Based on our NCBI database search, we found that LIN7A expression was significantly enriched in bone marrow tissues of normal subjects compared to other organs, suggesting its potential involvement in bone development (Figure 7A). This observation is consistent with our previous findings where we reported a downregulation of LIN7A expression during fracture non‐healing, as demonstrated in the GSE213891 database. To further investigate LIN7A expression in patients with bone non‐union, we collected blood samples from individuals at the Department of Orthopaedics, Chenggong Hospital of Xiamen University and performed RT‐qPCR to assess the expression levels of LIN7A and miR‐29b‐3p. Relevant clinical characteristics of each group, such as gender, age and postoperative time results were documented in Table S7. Blood samples were collected from patients in a fasting state before surgery. Our RT‐qPCR analysis revealed a significant reduction of LIN7A expression in patients with bone non‐union (Figure 7B), while miR‐29b‐3p transcript levels were also found to be downregulated (Figure 7C). Furthermore, our correlation analysis showed a positive association between LIN7A mRNA levels and miR‐29b‐3p expression in non‐union patients (Figure 7E). By performing ELISA to detect LIN7A protein levels in patients' serum, we observed a significant decrease in LIN7A expression in patients with bone non‐union (Figure 7D). Taken together, these results suggest that miR‐29b‐3p may play an inhibitory role in the healing process after fracture by regulating the expression of LIN7A protein. Our findings provide new insights into the molecular mechanisms underlying fracture non‐healing and may lead to the development of novel therapeutic strategies for this condition.

## 4 DISCUSSION

The success of bone healing depends on the coordinated interaction of biological and mechanical factors, and complications such as osteonecrosis can have a significant impact on patient prognosis.^3^, ^37^, ^38^ Although gene therapy represents a promising avenue for the development of new treatments and prognostic tools for various diseases, there is currently a lack of genomic studies focused on fracture healing.^1^, ^3^ Our study aims to fill this gap by investigating the genetic and molecular factors involved in bone healing, with the goal of identifying core genes and BPs that can serve as a foundation for future research.

miRNAs, small non‐coding RNA molecules, have been shown to play critical roles in various disease mechanisms and may serve as targets for novel therapeutic interventions in conditions such as osteoarthritis, bone cancer and bone discontinuity.^39^, ^40^, ^41^ For example, miR‐10a‐5p can induce apoptosis in chondrocytes by targeting HOXA1,^42^ while miRNA‐26a‐5p derived from M2 macrophages can promote osteogenic differentiation of bone mesenchymal stem cells.^43^ Several miRNAs, including miR‐31a‐5p,^44^ miR‐221^45^, ^46^ and miR‐451‐5p^47^ have also been identified as potential biomarkers in bone non‐union. However, research on miRNAs involved in bone non‐union is scarce compared to that on physiological bone healing. miR‐29 family is a common miRNA family involved in various diseases, including osteoporosis and osteoarthritis.^48^ MiR‐29 has been shown to promote osteoblast differentiation and apoptosis by suppressing target genes, while inhibiting chondrogenic differentiation, osteoclast differentiation, fibrosis and T‐cell differentiation.^48^, ^49^ The downregulation of miR‐29a/b/c expression in chondrocytes by TGF‐β1 ligands and phosphorylated Smad2/3 suggests its role in the pathogenesis of osteoarthritis.^50^

LIN7A, a small scaffolding protein containing the L27 structural domain, is essential for cell polarity, adhesion and signalling.^23^, ^24^ It stabilizes PALS1 and interacts with several MAGUK proteins through its PDZ structural domain.^51^ Our analysis shows that LIN7A expression is downregulated in non‐healing bone, suggesting its potential as a clinical marker of skeletal non‐healing. In addition, we found that LIN7A protein is highly expressed in normal bone marrow, suggesting a beneficial role in bone development.

In our study, our primary goal was to explore the molecular mechanisms that influence bone non‐union. We sought to identify more specific biomarkers associated with bone non‐union. To accomplish this, we performed differential expression analysis using bone non‐union‐related data from the GEO database. In addition, we used the WGCNA method to identify characteristic genes within network modules and cross‐referenced them with DEGs to pinpoint genes with a more precise impact on bone non‐union. Our exploration extended to understanding the biological functions affected by these key genes. Through GO enrichment analysis, we discovered that these genes were primarily related to cytokine and inflammatory factor secretion, endocytosis vesicle phagocytosis, and trans‐Golgi network regulation. Subsequently, using a bioinformatics approach, we tentatively concluded that miR‐29b‐3p may negatively affect fracture healing by modulating LIN7A expression. To validate this preliminary conclusion, we performed expression and correlation verification between miR‐29b‐3p and LIN7A in clinical samples using qPCR and ELISA. Ultimately, our results confirmed that miR‐29b‐3p downregulates LIN7A expression through targeted binding, thereby influencing the healing process in patients with bone non‐union.

However, our study has several limitations. First, the data we selected were derived from the GEO database, which lacked genomic studies on this topic and had a limited sample size for each sequencing data. Future studies will need to recruit larger sample sizes. Second, we found very limited samples and sequencing data, and additional samples would provide more analysis and validation and could be used to construct prognostic features. Finally, although we validated that miRNA‐29b‐3p plays a role in osteogenesis imperfecta and is able to regulate LIN7A expression in patients with osteogenesis imperfecta, we did not perform more detailed in vitro validation of the regulatory correlation between miRNA‐29b‐3p and LIN7A. In addition, we lacked cellular and animal experiments to investigate the regulatory role of LIN7A on downstream signalling pathways. Further experiments would help to elucidate the role of miRNA‐29b‐3p and LIN7A in the mechanism of skeletal non‐healing.

This study provides a thorough bioinformatic evaluation of genes that may be associated with the progression of bone non‐union. Furthermore, our findings suggest that LIN7A can be affected by miRNA‐29b‐3p, resulting in skeletal non‐healing. These findings may improve our understanding of the causes and basic molecular mechanisms of bone non‐union and thus provide valuable insights into its pathogenesis.

## 5 CONCLUSION

The overarching data demonstrated through bioinformatics and basic biology that downregulation of miRNA‐29b‐3p reduces the expression of LIN7A protein, which in turn affects the healing process after fracture in patients with bone non‐union, providing evidence for the accurate diagnosis and treatment of bone non‐union.

## AUTHOR CONTRIBUTIONS

**Guojian Jian:** Data curation (lead); methodology (lead); visualization (equal); writing – original draft (lead). **Desheng Xie:** Data curation (equal); formal analysis (equal). **Ximu Kuang:** Data curation (equal). **Peihuang Zheng:** Visualization (equal). **Haoyuan Liu:** Formal analysis (equal); software (equal). **Xuehong Dong:** Supervision (lead); writing – review and editing (equal).

## FUNDING INFORMATION

This work was supported by grants from NaturalScience Foundation of Xiamen, China (No. 3502Z202373123 2023), Xiamen Superior Sub‐specialty construction project of Arthroscopic minimally invasive Orthopaedics department (No. 2018296) and Xiamen Key specialty construction project of Traumatic Orthopaedics department (No. 2015347).

## CONFLICT OF INTEREST STATEMENT

The authors declare no competing interests.

## Supporting information


Figure S1.

Figure S2.

Figure S3.

Figure S4.

Figure S5.

Table S1.

Table S2.

Table S3.

Table S4.

Table S5.

Table S6.

Table S7.

## Data Availability

The data that supports the findings of this study are available in the supplementary material of this article. The data that support the findings of this study are available from the corresponding author upon reasonable request. The data that support the findings of this study are openly available in [repository name e.g “figshare”] at http://doi.org/[doi], reference number [10.1111/jcmm.18522.]
