# Supplementary figures and images for: Identification and validation of miR‐29b‐3p and LIN7A as important diagnostic markers for bone non‐union by WGCNA
Source: J Cell Mol Med. 2024 Jul 3;28(13):e18522. doi: 10.1111/jcmm.18522 (PMC11220363; doi:10.1111/jcmm.18522)

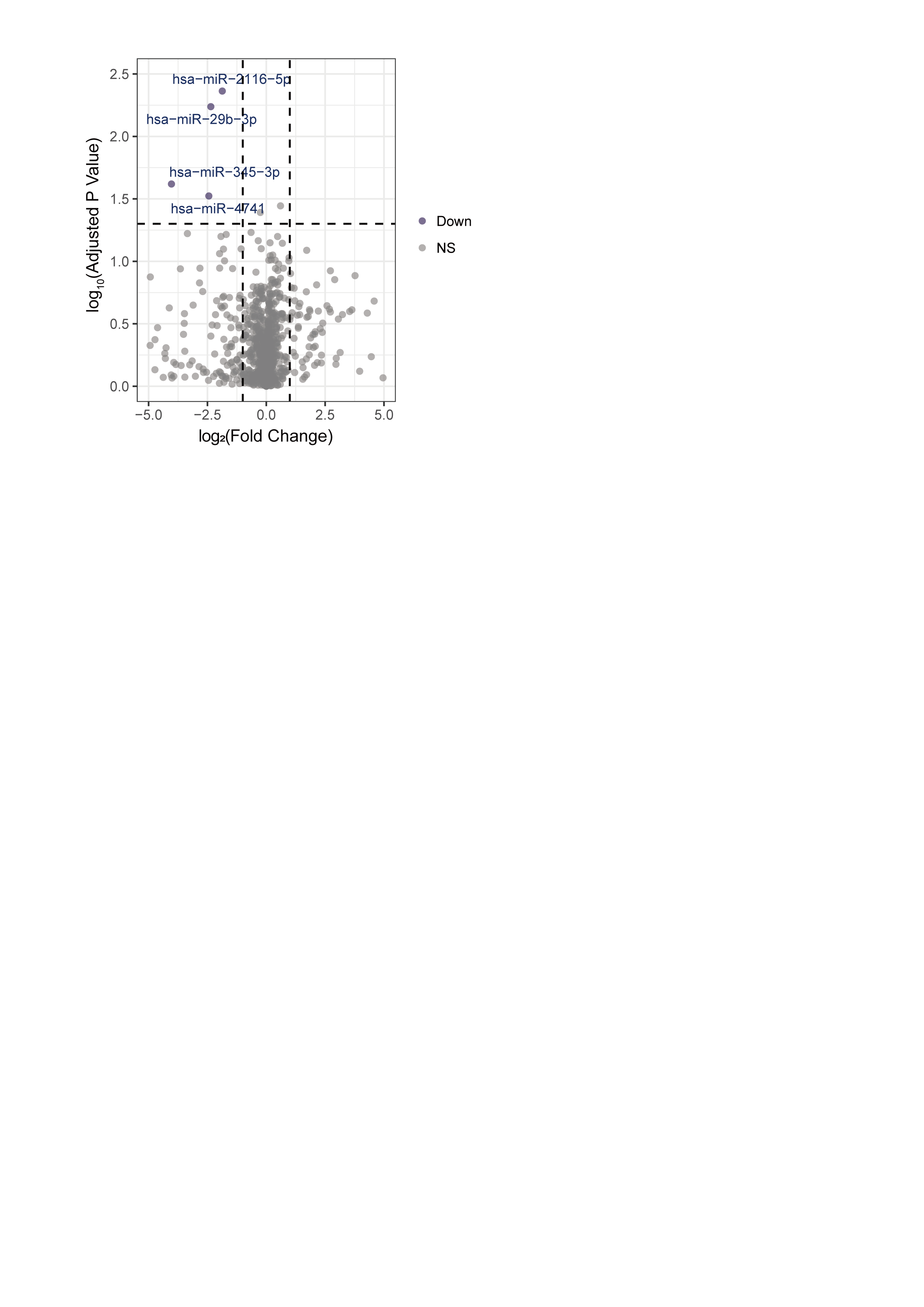

Supplement: Supplementary file 1 — Figure S1. [file JCMM-28-e18522-s011.tif]

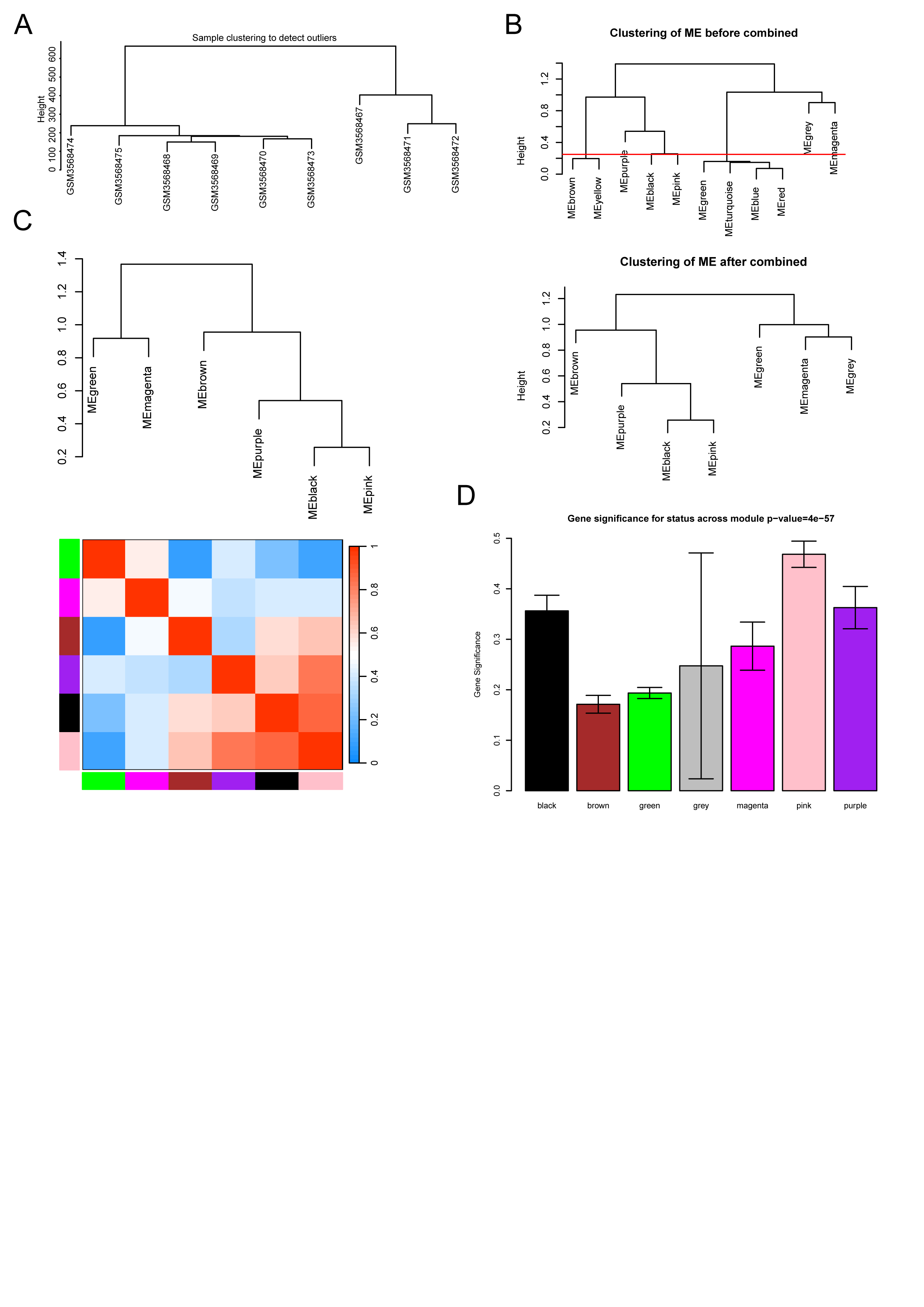

Supplement: Supplementary file 2 — Figure S2. [file JCMM-28-e18522-s010.tif]

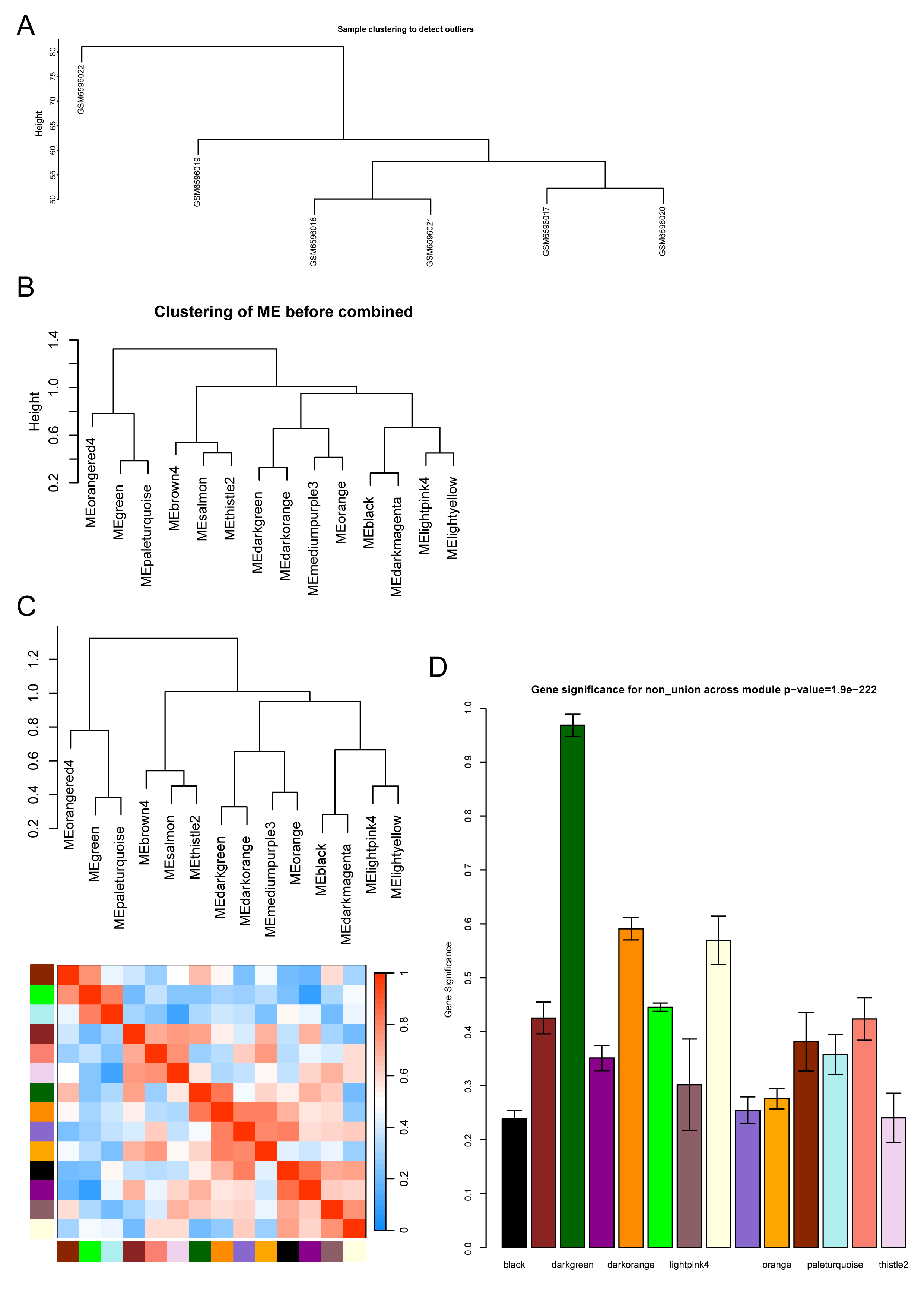

Supplement: Supplementary file 3 — Figure S3. [file JCMM-28-e18522-s006.tif]

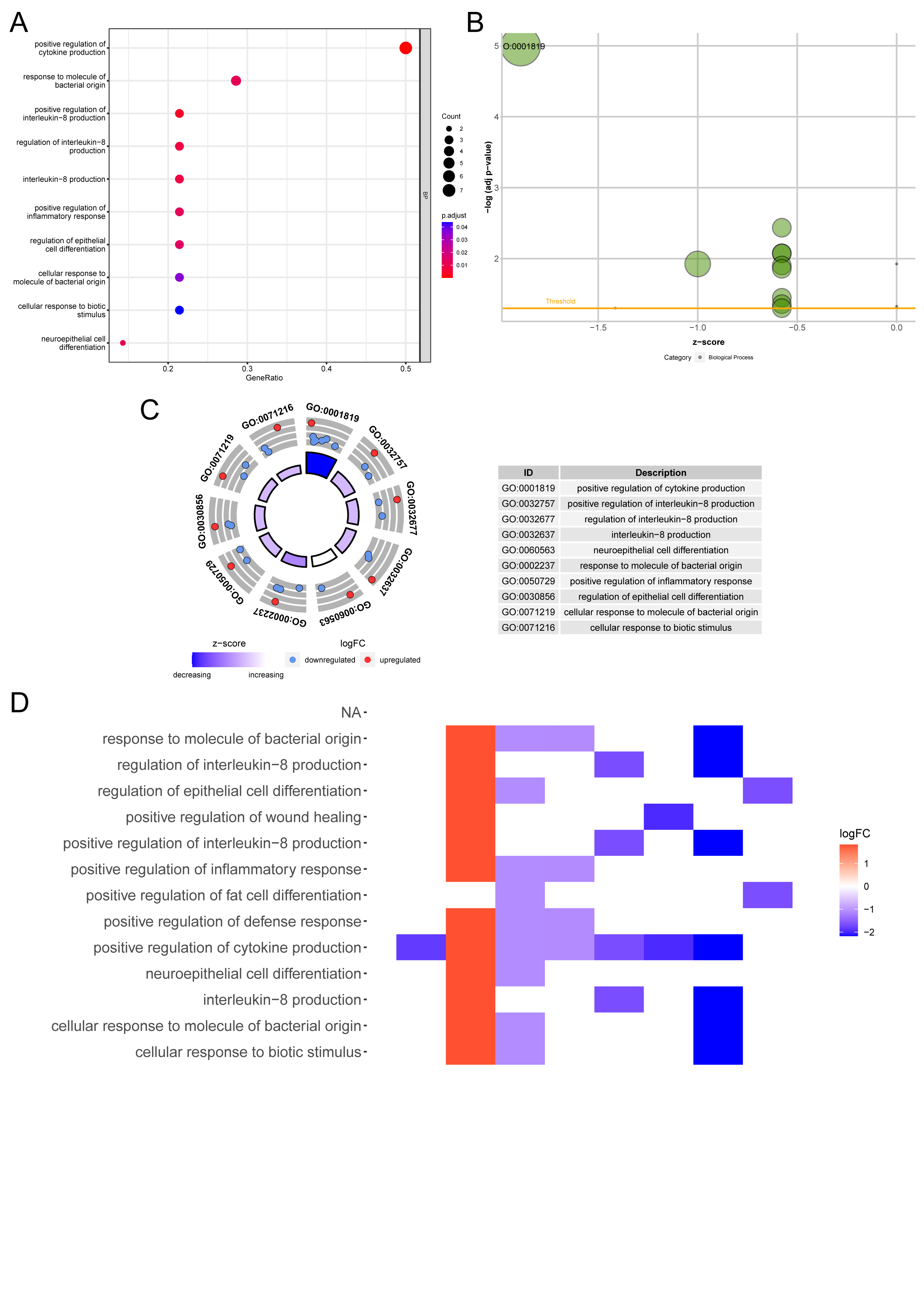

Supplement: Supplementary file 4 — Figure S4. [file JCMM-28-e18522-s002.tif]

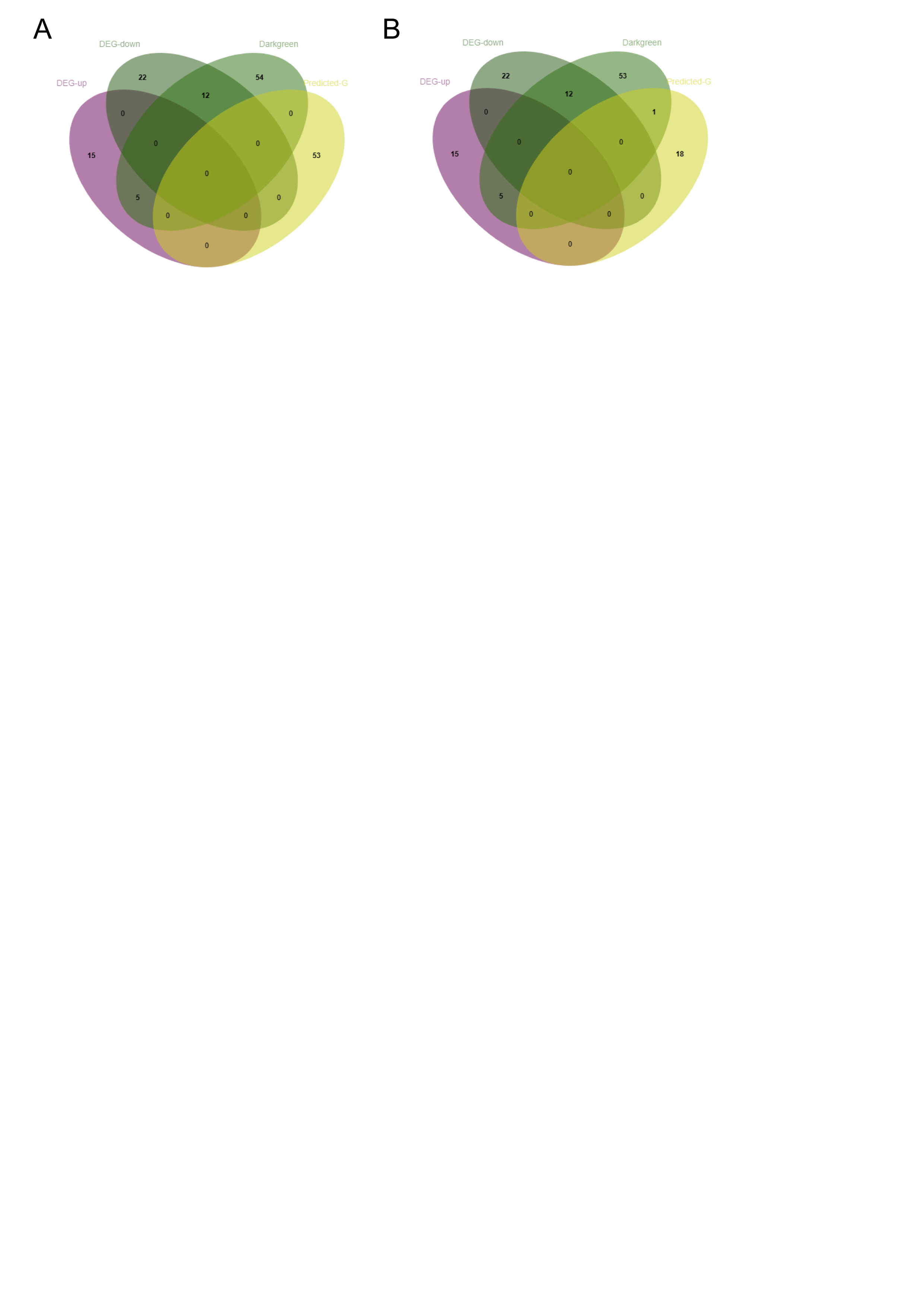

Supplement: Supplementary file 5 — Figure S5. [file JCMM-28-e18522-s005.tif]
